# Amyloid-beta peptide degradation in cell cultures by mycoplasma contaminants

**DOI:** 10.1186/1756-0500-1-38

**Published:** 2008-06-30

**Authors:** Haitian Zhao, Ute Dreses-Werringloer, Peter Davies, Philippe Marambaud

**Affiliations:** 1 Litwin-Zucker Research Center for the Study of Alzheimer Disease, The Feinstein Institute for Medical Research, North Shore-LIJ, Manhasset, NY, USA; 2 Department of Pathology, Albert Einstein College of Medicine, Bronx, NY, USA

## Abstract

**Background:**

Cell cultures have become an indispensable tool in Alzheimer's disease research for studying amyloid-β (Aβ) metabolism. It is estimated that up to 35% of cell cultures in current use are infected with various mycoplasma species. In contrast with common bacterial and fungal infections, contaminations of cell cultures with mycoplasmas represent a challenging issue in terms of detectability and prevention. Mycoplasmas are the smallest and simplest self-replicating bacteria and the consequences of an infection for the host cells are variable, ranging from no apparent effect to induction of apoptosis.

**Findings:**

Here we present evidence that mycoplasmas from a cell culture contamination are able to efficiently and rapidly degrade extracellular Aβ. As a result, we observed no accumulation of Aβ in the conditioned medium of mycoplasma-positive cells stably transfected with the amyloid-β precursor protein (APP). Importantly, eradication of the mycoplasma contaminant – identified as *M. hyorhinis *– by treatments with a quinolone-based antibiotic, restored extracellular Aβ accumulation in the APP-transfected cells.

**Conclusion:**

These data show that mycoplasmas degrade Aβ and thus may represent a significant source of variability when comparing extracellular Aβ levels in different cell lines. On the basis of these results, we recommend assessment of mycoplasma contaminations prior to extracellular Aβ level measurements in cultured cells.

## Findings

**Figure 1:**
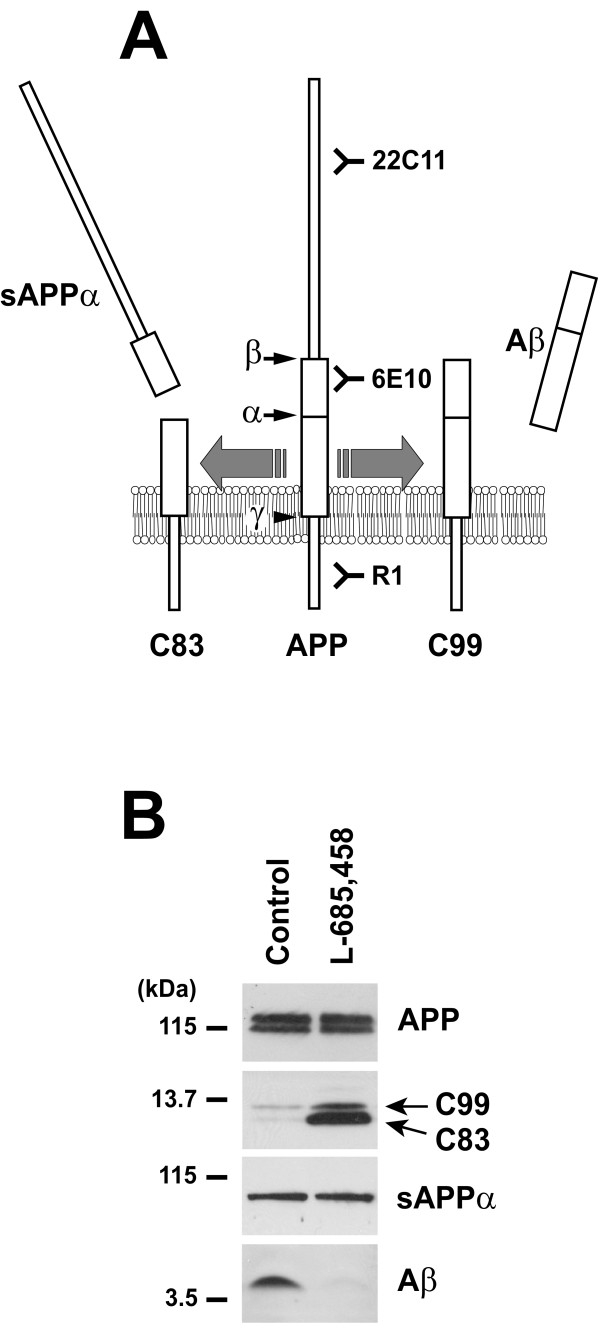
**APP processing**. (A) Schematic representation of APP processing. APP undergoes two alternative endoproteolytic pathways. In the amyloidogenic pathway, APP is first cleaved by β-secretase (β) to enable the production of the membrane bound C99 fragment. C99 is then cleaved in its intramembranous domain by γ-secretase (γ) to release Aβ. In the non-amyloidogenic pathway, APP is cleaved by α-secretase (α) to generate sAPPα and the membrane bound C83 fragment. Immunogenic regions for the indicated anti-APP antibodies are shown. (B) HEK293 cells stably transfected with Swedish human APP_695 _cDNA [16] were incubated in the absence (Control) or presence of γ-secretase inhibitor (L-685,458, Calbiochem, 1 μM for 18 hrs). APP full-length (APP) and C-terminal fragments (C99 and C83) were analyzed by western blotting (WB) as described before [16], using antibodies directed against APP N-terminal domain (22C11, Chemicon) and APP C-terminal domain (R1, provided by Dr. P.D. Mehta, see Ref. [16]), respectively. Secreted sAPPα and Aβ were analyzed by WB using anti-APP_1–17 _antibody (6E10, Signet). Wild type and APP-transfected HEK293 cells were grown in DMEM plus 10% FBS, penicillin and streptomycin in 5% CO_2 _at 37°C. APP-transfected HEK293 cells were selected and maintained in 5 μg/ml puromycin.

Alzheimer's disease (AD) is a progressive neurodegenerative disorder characterized by the presence of classical lesions in different brain regions of the neocortex and hippocampus [1]. Among these lesions, the amyloid plaques formed by the aggregation of amyloid-β (Aβ) peptides are prominent. The pathogenesis of the disease is complex and is driven by both environmental and genetic factors. Although most of the cases are sporadic with an obscure etiology, some forms of the disease are inherited and several genes are implicated in familial Alzheimer's disease (FAD). The understanding of the molecular basis of the disease gained significant knowledge with the observation that mutations in the three genes linked to early-onset autosomal dominant FAD, increase the production of a highly insoluble isoform of Aβ. Together, these observations support the so-called amyloid hypothesis in the pathogenesis of Alzheimer's disease [2,3]. Sequential endoproteolysis of the amyloid-β precursor protein (APP) by the aspartic protease β-secretase/BACE1 and by the γ-secretase proteolytic complex leads to the production of Aβ (see Figure 1A). In an alternative non-amyloidogenic pathway, APP is endoproteolyzed within the Aβ region by α-secretase to produce the secreted sAPPα fragment (Figure 1A) [4,5].

Numerous primary and immortalized cell lines have been used to analyze APP processing and Aβ production in *in vitro *cultures. These cell culture systems have proven to be indispensable for the identification of pharmacological and genetic modifiers of APP metabolism prior to *in vivo *studies [6-8]. It is estimated that 15 to 35% of cell cultures in current use are infected with mycoplasmas [9]. With a diameter of about 0.2 – 0.4 μm, mycoplasmas are small, slow-growing bacteria that are generally unaffected by the antibiotics used against common bacteria and fungi. They can go undetected for long periods of time as the contaminated cells may grow normally and appear normal by light microscopy. Mycoplasma contaminations can however have negative effects, ranging from inhibition of metabolism and growth to induction of malignant transformation or apoptosis [10,11]. In this study, we show that mycoplasmas can degrade extracellular Aβ. These results indicate that mycoplasma contaminations can introduce an unsuspected source of variability in Aβ level measurements in cultured cells.

### Characterization of extracellular Aβ produced by APP-transfected cells

The APP metabolites, sAPPα and Aβ, are readily detectable in the conditioned medium of HEK293 cells stably transfected with the human APP_695 _isoform (Figure 1B, first lane). As expected, treatment of these cells with the selective γ-secretase inhibitor, L-685,458, prevented Aβ production and promoted accumulation of the APP intermediate fragments C99 and C83 (Figure 1B, second lane).

### Degradation of extracellular Aβ by mycoplasma contaminants

**Figure 2:**
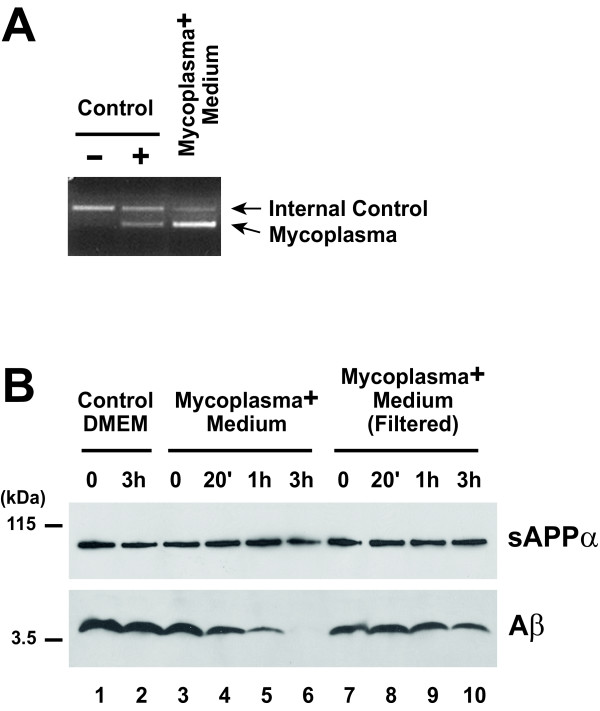
**Degradation of extracellular Aβ by mycoplasma contaminants**. (A) PCR amplification of mycoplasma genomic DNA was performed with an internal negative control template (Control -), a positive control template of *M. orale *genomic DNA (Control +), or with medium from mycoplasma-positive HEK293 cell cultures (Mycoplasma+ Medium). Assay was carried out according to manufacturer's instructions (MycoSensor PCR Assay Kit, Stratagene). The changes in intensity of the internal control amplification are due to competition with the mycoplasma-specific PCR. (B) Medium from mycoplasma-positive cells was centrifuged at 1000 × g for 10 min to pellet the cell debris. Conditioned medium from APP-transfected HEK293 cells was diluted 1:1 with either fresh medium (Control DMEM), or with unfiltered (Mycoplasma+ Medium) or 0.2 μm filtered [Mycoplasma+ Medium (Filtered)] medium from mycoplasma-positive cells. The resulting mixtures were incubated at 37°C for the indicated periods of time. sAPPα and Aβ were analyzed by WB, as described in Figure 1B.

To first assess the effect of mycoplasmas on the levels of Aβ in cell culture, medium from mycoplasma-positive cells, detected by polymerase-mediated amplification of mycoplasma genomic DNA (Figure 2A), was diluted 1:1 with conditioned medium from APP-transfected HEK293 cells. The resulting mixture was then incubated at 37°C for different periods of time. Under these conditions, we observed a robust degradation of Aβ after 1 hr of incubation, while sAPPα levels were not affected (Figure 2B, lanes 3–6). Aβ degradation did not occur when fresh medium (Control DMEM) was mixed with Aβ-containing medium (Figure 2B, lanes 1 and 2). Importantly, reduction of the contaminant load by a 0.2 μm filtration of the mycoplasma-positive medium prior to incubation, significantly prevented Aβ degradation (Figure 2B, lanes 7–10). Together these data strongly indicate that mycoplasmas efficiently degrade Aβ during normal cell culture conditions.

### Mycoplasma contamination prevents Aβ accumulation in APP-transfected cells

**Figure 3:**
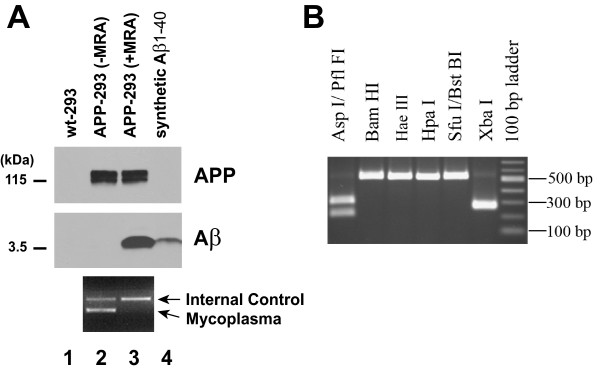
**Mycoplasma eradication restored Aβ accumulation in APP-transfected cells**. (A) APP (first panel) and Aβ (second panel) levels in wild-type (wt-293) and APP-transfected (APP-293) HEK293 cells were analyzed by WB, as described before. APP-293 cells were cultured for one week in the absence (-MRA) or presence (+MRA) of Mycoplasma Removal Agent (MRA, MP Biomedicals, Inc). Conditioned medium of untreated and treated APP-293 cells was analyzed by PCR for the presence of mycoplasma genomic DNA (lower panel), as described above. Twenty-five picograms of synthetic Aβ1–40 (IBL-America) were loaded as a control (lane 4). (B) Determination of the mycoplasma species by restriction fragment polymorphism analysis of PCR products. The PCR product was digested with six different restriction endonucleases as indicated and separated on an agarose gel. The fragment pattern identifies the contaminant as *M. hyorhinis*. Briefly, a PCR reaction is performed with a mix of 9 primers to cover a range of mycoplasma species, including major contaminants of cell cultures. The 500–520 bp PCR products, depending on the species, are then subjected to several digests with restriction endonucleases to differentiate between 7 mycoplasma species, as previously described [14]. Cells were cultured without antibiotic for several days. 1 ml of supernatant and 1 ml of trypsinized cells were centrifuged for 10 min at 13,000 rpm and DNA was extracted using DNeasy Blood and Tissue Kit (Qiagen). The PCR was carried out in 50 μl containing 0.2 mM of each deoxynucleotide, 0.2 μM of each primer (sequences provided in Ref. [17]), 1.5 mM MgCl_2_. PCR conditions were as follows, 5 min denaturation at 95°, 35 cycles of 30 sec at 95°C, 30 sec annealing at 65°C and 1 min extension at 72°C followed by a final extension for 10 min. 5 μl of the PCR products were used for restriction digests in 20 μl of reaction volume for 3 hrs. The following enzymes were used: Pfl FI (isoschizomer of Asp I), Bam HI, Hae III, Hpa I, BstBI (isoschizomer of Sfu I) and Xba I. Fragments were analyzed on a 1.5% agarose gel.

In order to confirm the detrimental effect of mycoplasma contamination on Aβ accumulation in cell culture, we directly analyzed Aβ levels produced by mycoplasma-positive APP-transfected HEK293 cells. Interestingly, no Aβ was detected in the conditioned medium of the mycoplasma-positive cells (Figure 3A, lane 2), while these cells strongly overexpressed APP (Figure 3A, upper panel, lanes 1 and 2).

### Mycoplasma eradication restored Aβ accumulation in APP-transfected cells

Successful mycoplasma decontamination can be achieved by treatments with different antibiotics, including quinolones and tetracyclines [9]. Contaminated APP-transfected cells were treated for one week with the quinolone-based antibiotic, MRA (4-oxo-quinolone-3-carboxylic acid derivative). The antibiotic treatment successfully eradicated the mycoplasmas (Figure 3A, lower panel), and restored Aβ accumulation in the conditioned medium of the APP-transfected cells (Figure 3A, middle panel). *M. hyorhinis*, *M. orale*, *M. arginini*, *M. fermentans*, *M. hominis *and *Acholeplasma laidlawii *represent 90–95% of the contaminants in mycoplasma-positive cells [12,13]. By restriction fragment length polymorphism assay of PCR products amplified from a region of the conserved 16S rDNA gene in mycoplasma species [14], we determined that the APP-transfected cells were contaminated with the *M. hyorhinis *species (Figure 3B). Together these results show that mycoplasmas degrade Aβ in cell cultures.

## Discussion

Aβ peptides are degraded in cell culture systems and *in vivo *by at least four peptidases from the metallopeptidase family, neprilysin, endothelin-converting enzyme-1 and -2, and insulin-degrading enzyme [15]. Bacteria express numerous proteases with important biological activities, such as degradation of tissue matrix proteins or activation of zymogens through limited proteolysis. The vast majority of the contaminations in mycoplasma-positive cells are due to *M. hyorhinis*, *M. orale*, *M. arginini*, *M. fermentans*, *M. hominis *and *Acholeplasma laidlawii *[12,13]. Partial sequencing of the *M. hominis *genome predicted the expression of at least three metallopeptidases, and *M. penetrans*, a mycoplasma species isolated from human, is predicted to express oligopeptidase O1, a metallopeptidase from the M13 neprilysin family. Here *M. hyorhinis *was identified in the contaminated APP-transfected cell lines. It is conceivable that *M. hyorhinis *also expresses metallopeptidases with homologies with known mammalian metallopeptidases, which degrade Aβ but not large secreted proteins, such as sAPPα. Contamination with mycoplasmas would therefore provide a very efficient peptidase-driven mechanism of Aβ clearance.

In conclusion, we show that (i) mycoplasmas from cell culture contaminations degrade *in vitro *Aβ produced in cell lines, (ii) mycoplasma-positive APP-transfected cells do not accumulate Aβ in the conditioned medium, and (iii) eradication of the mycoplasma contaminant (i.e. *M. hyorhinis*) by treatments with a quinolone-based antibiotic, restored extracellular Aβ accumulation in APP-transfected cells. Together these results demonstrate that mycoplasmas may represent a significant source of variability when comparing extracellular Aβ levels between different cell lines. We therefore recommend assessment of mycoplasma contamination prior to extracellular Aβ level measurements in cultured cells.

## Competing interests

The authors declare that they have no competing interests.

## Authors' contributions

HZ, UDW, and PM performed experiments. PD and PM discussed the experimental strategy. PM and UDW wrote the manuscript.

## References

[B1] Selkoe DJ (2001). Alzheimer's disease: genes, proteins, and therapy. Physiol Rev.

[B2] Hardy J, Selkoe DJ (2002). The amyloid hypothesis of Alzheimer's disease: progress and problems on the road to therapeutics. Science.

[B3] Golde TE (2005). The Abeta hypothesis: leading us to rationally-designed therapeutic strategies for the treatment or prevention of Alzheimer disease. Brain Pathol.

[B4] Checler F (1995). Processing of the beta-amyloid precursor protein and its regulation in Alzheimer's disease. J Neurochem.

[B5] Marambaud P, Robakis NK (2005). Genetic and molecular aspects of Alzheimer's disease shed light on new mechanisms of transcriptional regulation. Genes Brain Behav.

[B6] Weggen S, Eriksen JL, Das P, Sagi SA, Wang R, Pietrzik CU, Findlay KA, Smith TE, Murphy MP, Bulter T, Kang DE, Marquez-Sterling N, Golde TE, Koo EH (2001). A subset of NSAIDs lower amyloidogenic Abeta42 independently of cyclooxygenase activity. Nature.

[B7] Wolfe MS, Xia W, Ostaszewski BL, Diehl TS, Kimberly WT, Selkoe DJ (1999). Two transmembrane aspartates in presenilin-1 required for presenilin endoproteolysis and gamma-secretase activity. Nature.

[B8] Xia X, Wang P, Sun X, Soriano S, Shum WK, Yamaguchi H, Trumbauer ME, Takashima A, Koo EH, Zheng H (2002). The aspartate-257 of presenilin 1 is indispensable for mouse development and production of beta-amyloid peptides through beta-catenin-independent mechanisms. Proc Natl Acad Sci U S A.

[B9] Uphoff CC, Drexler HG (2002). Comparative antibiotic eradication of mycoplasma infections from continuous cell lines. In Vitro Cell Dev Biol Anim.

[B10] Tsai S, Wear DJ, Shih JW, Lo SC (1995). Mycoplasmas and oncogenesis: persistent infection and multistage malignant transformation. Proc Natl Acad Sci U S A.

[B11] Sokolova IA, Vaughan AT, Khodarev NN (1998). Mycoplasma infection can sensitize host cells to apoptosis through contribution of apoptotic-like endonuclease(s). Immunol Cell Biol.

[B12] Hay RJ, Macy ML, Chen TR (1989). Mycoplasma infection of cultured cells. Nature.

[B13] Drexler HG, Uphoff CC (2000). Contamination of cell culture, mycoplasma. Encyclopedia of cell technology Volume I Edited by : Spier RE New York, John Wiley & Sons, Inc.

[B14] Uphoff CC, Drexler HG (2002). Comparative PCR analysis for detection of mycoplasma infections in continuous cell lines. In Vitro Cell Dev Biol Anim.

[B15] Turner AJ, Fisk L, Nalivaeva NN (2004). Targeting amyloid-degrading enzymes as therapeutic strategies in neurodegeneration. Ann N Y Acad Sci.

[B16] Marambaud P, Zhao H, Davies P (2005). Resveratrol promotes clearance of Alzheimer's disease amyloid-beta peptides. J Biol Chem.

[B17] Wirth M, Berthold E, Grashoff M, Pfutzner H, Schubert U, Hauser H (1994). Detection of mycoplasma contaminations by the polymerase chain reaction. Cytotechnology.

